# The evaluation of exposure risks for natural transmission of scrapie within an infected flock

**DOI:** 10.1186/1746-6148-5-38

**Published:** 2009-10-09

**Authors:** Glenda Dexter, Sue C Tongue, Lindsay Heasman, Susan J Bellworthy, Andrew Davis, S Jo Moore, Marion M Simmons, A Robin Sayers, Hugh A Simmons, Danny Matthews

**Affiliations:** 1 Veterinary Laboratories Agency, Woodham Lane, New Haw, Addlestone, Surrey KT15 3NB, UK; 2 ADAS, High Mowthorpe, Duggleby, Malton, North Yorkshire YO17 8BP, UK; 3 Veterinary Medicines Directorate, Woodham Lane, New Haw, Addlestone, Surrey, KT15 3LS, UK; 4 Westpoint Veterinary Group, Dawes Farm, Bognor Road, Warnham, West Sussex, RH12 3SH, UK; 5 School of Veterinary Science, University of Queensland, Pathology, Infectious Disease & Biosecurity, St Lucia, Australia

## Abstract

**Background:**

Although the epidemiology of scrapie has been broadly understood for many years, attempts to introduce voluntary or compulsory controls to eradicate the disease have frequently failed. Lack of precision in defining the risk factors on farm has been one of the challenges to designing control strategies. This study attempted to define which parts of the annual flock management cycle represented the greatest risk of infection to naive lambs exposed to the farm environment at different times.

**Results:**

In VRQ/VRQ lambs exposed to infected sheep at pasture or during lambing, and exposed to the buildings in which lambing took place, the attack rate was high and survival times were short. Where exposure was to pasture alone the number of sheep affected in each experimental group was reduced, and survival times were longer and related to length of exposure.

**Conclusion:**

At the flock level, eradication and control strategies for scrapie must take into account the need to decontaminate buildings used for lambing, and to reduce (or prevent) the exposure of lambs to infected sheep, especially in the later stages of incubation, and at lambing. The potential for environmental contamination from pasture should also be considered. Genotype selection may still prove to be the only viable tool to prevent infection from contaminated pasture, reduce environmental contamination and limit direct transmission from sheep to sheep.

## Background

Attempts to unravel the epidemiology of scrapie have been frustrated by many factors including long incubation periods, lack of host immune response, difficulties associated with isolation and characterisation of the infectious agent, and the challenge of proving that animals which are exposed to natural or experimental infection are truly naive.

The association of disease with family lines at one time led to suggestions that the disease was entirely genetic [1], but subsequent studies in several breeds highlighted the fact that disease results from an interaction between host genotype and the infectious agent [2-7]. In recent years research into the relationship between genotype and susceptibility to infection or disease has expanded considerably [8] and highlighted the influence of three codons of the prion protein gene (136, 154, 171). Such findings have resulted in their use, on a precautionary basis, in structuring voluntary and compulsory breeding programmes intended to reduce the risk to consumers from the potential presence of BSE in sheep [9-11].

Reviews [12-14] re-iterate the basic assumptions of scrapie epidemiology: transmission between farms is primarily associated with the movement of infected animals [15,16], although a role for contaminated feed has also been postulated [17]; once introduced into a flock, infection can be transmitted both maternally from mother to lamb, and horizontally to unrelated in-contact sheep [16,18-23]. A seasonal risk associated with lambing has been predicted in an experimental scrapie flock at INRA, France [23], but modelling of the scrapie outbreak in the INRA flock could not exclude horizontal transmission at other times. Greater precision in the identification of risk areas and risk periods should assist the development of intervention strategies following the diagnosis of scrapie in a flock.

It is difficult to prove the naive status of experimental animals prior to exposure, therefore many studies established in the United Kingdom since 1998 have relied upon sheep imported from New Zealand. These sheep are considered to be free of classical scrapie.

The introduction of such naive dams and their lambs into an experimental flock in which scrapie is maintained naturally resulted in transmission to both [22]. Given that horizontal transmission is necessary to maintain scrapie within a flock [14,24], we investigated components of the management cycle within that flock (the Veterinary Laboratories Agency (VLA) scrapie flock), from lambing to grazing, with or without contact with infected sheep, to determine which, if any, represented the greatest risk. The main focus of this paper is the outcome of investigations in lambs of the VRQ/VRQ genotype.

## Results

### The VLA scrapie flock

**Figure 1:**
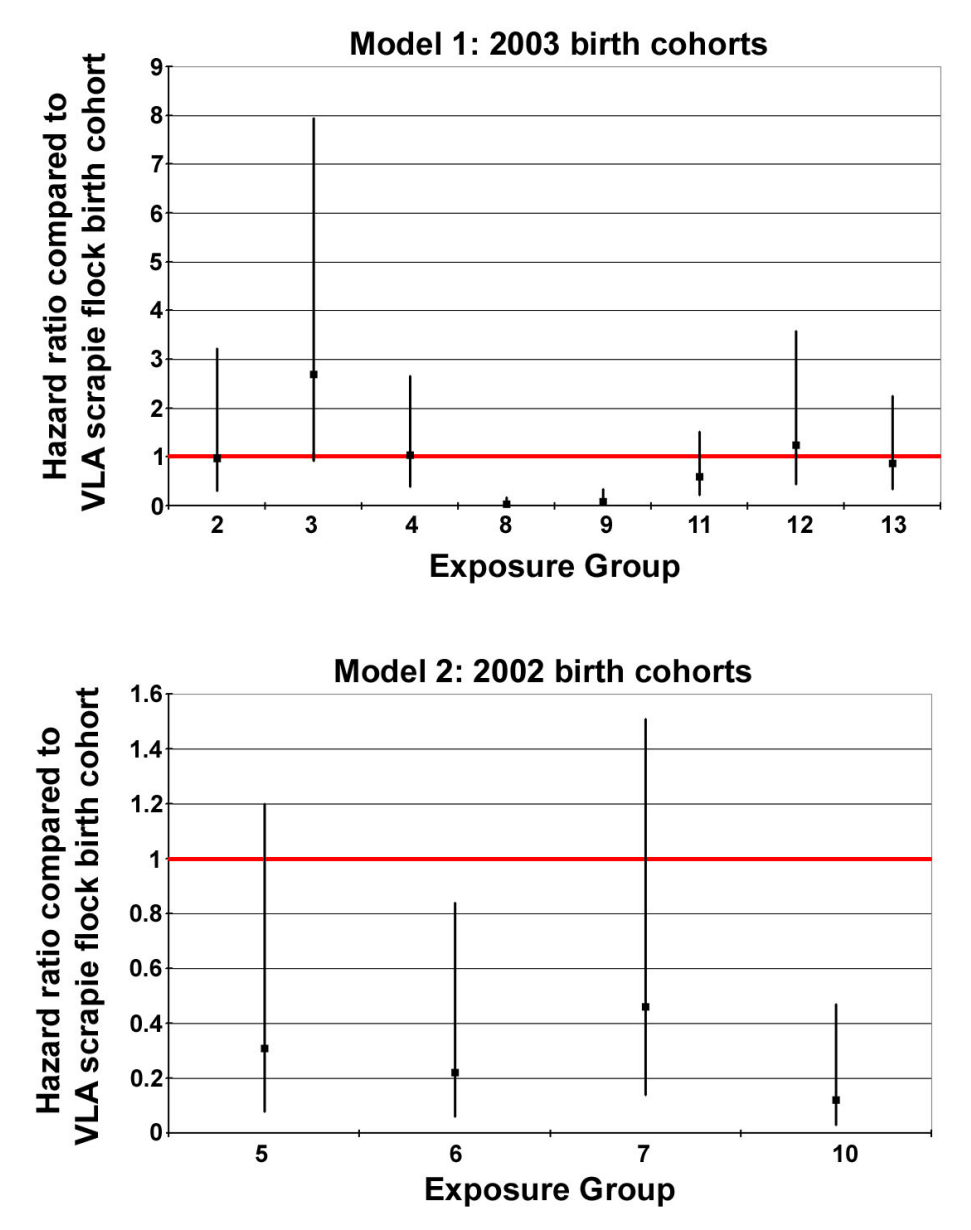
**Hazard ratios and 95% confidence intervals for survival time for the exposure groups compared to the baseline birth cohort (red line)**. Model 1: the baseline is the naturally exposed VLA scrapie flock 2003 birth cohort. Model 2: the baseline is the naturally exposed VLA scrapie flock 2003 birth cohort.

Within the VLA scrapie flock there was evidence for significantly longer survival times in the 2003 birth cohort, compared to the 2002 birth cohort (Hazard Ratio 2.779, *P *= 0.023). Seven of the eight VRQ/VRQ lambs which were born in 2002 and remained in the flock until death developed clinical disease and were killed between 592 and 730 days of age (median = 709 days) (Table 1). Numbers in this group were limited by the high rate of withdrawal of lambs in that year to service other projects. Forty one of 46 VRQ/VRQ lambs born in the 2003 birth cohort developed clinical disease and were killed between 601 and 1184 days (median = 748 days) (Table 1). In light of these differences the experimental groups were analysed separately by birth cohort using the relevant VLA scrapie flock birth cohort as the baseline for comparison (Table 2, Figure 1).

**Table 1:** Data for experimental exposure groups and VLA scrapie flock cohorts.

**Exposure Group**	**Birth cohort**	**Dam status**	**Area(s) exposed to**	**Length of time exposed to infected environment**	**Type of contact with VLA scrapie flock sheep**	**Number in group at start of exposure**	**Number of clinical cases**	**Median** **survival time (days)**	**Range survival time (days)**
1	2003	N	None	N/A	None	7	0	-	-
2	2003	N	All	12 months	Direct	4	3*	792	747-792
3	2003	N	Lambing pens	6 weeks	Direct	6	4*	730	663-742
4	2003	N	Lambing pens	6 weeks	Indirect	6	5*	770	709-836
5	2002	A	All	6 weeks	Direct	5	5	659	639-857
6	2002	A	All	3 months	Direct	5	5	780	644-857
7	2002	A	All	12 months	Direct	5	5	728	716-775
8	2003	N	Pasture	6 weeks	Indirect	5	2	-	1094-1673
9	2003	N	Pasture	3 months	Indirect	5	4	1299	836-1299
10	2002	N	Pasture	12 months	Indirect	8	6*	794	729-869
11	2003	N	Pasture	6 weeks	Direct	5	5	817	728-1008
12	2003	N	Pasture	3 months	Direct	5	4*	776	684-791
13	2003	N	Pasture	12 months	Direct	5	5	807	742-823
VLA scrapie flock	2002	A	All	Birth to clinical endpoint	Direct	8	7*	709	592-730
VLA scrapie flock	2003	A	All	Birth to clinical endpoint	Direct	46	41*	748	601-1184

Dam status - N, naïve, TSE-free; A, scrapie affected.

* 'Number of clinical cases' is different to 'Number in group at start of exposure' due to intercurrent deaths.

**Table 2:** Cox proportional hazard models for the exposure groups by birth cohort, with the naturally exposed VLA scrapie flock birth cohorts as the baseline categories.

**Exposure group**	**Hazard Ratio**	**95% C.I.**	**P-value**
**Model 1**			

2003 birth cohort	Baseline		
2	0.98	0.30 - 3.22	0.969
3	2.70	0.92 - 7.95	0.071
4	1.04	0.40 - 2.66	0.942
8	0.03	0.005 - 0.17	**< 0.001**
9	0.09	0.02 - 0.34	**< 0.001**
11	0.60	0.23 - 1.52	0.279
12	1.25	0.44 - 3.58	0.680
13	0.87	0.34 - 2.25	0.774
Overall model			**< 0.001**

**Model 2**			

2002 birth cohort	Baseline		
5	0.31	0.08 - 1.20	0.091
6	0.22	0.06 - 0.84	**0.027**
7	0.46	0.14 - 1.51	0.198
10	0.12	0.03 - 0.47	**0.002**
Overall model			**0.032**

In an effort to better understand the differences between the 2002 and 2003 VLA scrapie flock cohorts we also analysed data on the frequency of clinical scrapie in the flock as a whole since its creation in 1998.

The size of the VLA scrapie flock has varied from 68 in 1998 to 703 at the time of this study. Across all genotypes the number of cases of clinical scrapie has ranged between eight in 2001, most probably the first home-bred cases, to 81 in 2007. Numbers of clinical cases have varied in part due to the maturation of the flock after its establishment from known scrapie-affected sources, but also due to the removal of sheep to other projects during the preclinical stages of disease. In 2002 and 2003, total clinical case numbers were 51 and 35 respectively.

The overall crude invariable incidence rate of clinical scrapie in the VLA scrapie flock between its establishment in 1998 and 31^st ^August 2007 was nine cases per 100 sheep years at risk. For VRQ/VRQ sheep the crude incidence rate was 33 per 100 sheep years at risk (95% confidence interval, 29 to 38), and for ARQ/ARQ sheep was 2.7 per 100 sheep years at risk (95% confidence interval, 1.7 to 44). Taking the incidence of clinical scrapie in ARQ/ARQ sheep as a baseline, VRQ/VRQ sheep are 12 times more likely to develop clinical disease (95% confidence interval, 8 to 20).

### Experimental Groups

The 15 positive control lambs (Groups 5, 6 and 7) born to scrapie-affected ewes developed clinical disease and were killed between 638 and 865 days of age (Table 1). The survival times of Groups 5-7 were not significantly different to each other (*P *= 0.552) or to the 2002 VLA scrapie flock cohort (Table 2, Model 2) except for Group 6, which had significantly shorter survival times than the 2002 VLA scrapie flock cohort (*P *= 0.027).

None of the negative control sheep (Group 1) died of scrapie after an observation period of 1795 days (Table 1).

All animals that had direct contact with the infected flock (Groups 2, 3, 11, 12 and 13, all born in 2003) reached clinical end-point and were killed with survival times of 663-1008 days (Table 1). Survival times for these groups were not significantly different to each other (Table 3) or the 2003 VLA scrapie flock cohort (Table 2, Model 1), with the exception of Group 11, exposed to pasture for six weeks, which had significantly longer survival times than Group 3 (*P *= 0.031), exposed to lambing environment for six weeks.

**Table 3:** Pair-wise comparison of VLA scrapie flock birth cohorts and exposure groups born in the same year.

	**Exposure Group**											
**Exposure Group**	**2**	**3**	**4**	**5**	**6**	**7**	**8**	**9**	**10**	**11**	**12**	**13**

**2002 birth cohort**				0.091	**0.027**	0.198			**0.002**			

**5**					0.568	0.586			0.13			

**6**						0.28			0.344			

**7**									0.053			

**2003 birth cohort**	0.969	0.071	0.942				**< 0.001**	**< 0.001**		0.279	0.68	0.774

**2**		0.192	0.936				**0.001**	**0.008**		0.506	0.749	0.875

**3**			0.164				**< 0.001**	**< 0.001**		**0.031**	0.286	0.101

**4**							**< 0.001**	**0.003**		0.391	0.782	0.784

**8**								0.184		**0.002**	**< 0.001**	**< 0.001**

**9**										**0.017**	**0.002**	**0.005**

**11**											0.282	0.557

**12**												0.593

All six sheep in Group 4, exposed to the infected lambing environment with no direct contact with scrapie-affected sheep, died. Five of the six developed clinical disease with a median survival time of 770 days (Table 1). One died of intercurrent disease at 265 days post-exposure with no signs of infection. Survival times for Group 4 were not significantly different to the 2003 VLA scrapie flock cohort or Groups 2, 3, 11-13, but were significantly longer than Groups 8 and 9 (Table 3), exposed to pasture without direct contact with scrapie-affected sheep for six weeks and 12 months respectively.

Of the eight sheep in Group 10, exposed to infected pasture for twelve months with no direct contact with the infected flock, six died of scrapie with a median survival time of 794 days. The remaining two sheep died from intercurrent disease (Table 1). Survival times for this Group were significantly longer than for the 2002 VLA scrapie flock cohort (Table 2, Model 2) but were not different to the other three experimental groups born in the same year (Groups 5-7) (Table 3).

When the project was terminated at 1794 days post-exposure one of five sheep in Group 9, exposed to pasture for 122 days, was still alive, but four had died of clinical scrapie with a median incubation period of 1299 days. In Group 8, exposed to pasture for only 42 days, only two sheep had died of scrapie, at 1094 and 1673 days respectively (Table 1). Survival times for these two groups were not significantly different from each other, but both these groups had a statistically significant lower risk of failure, i.e. longer survival times, than the baseline 2003 VLA scrapie flock cohort (Table 2, Model 1) and all other experimental groups born in 2003 (Table 3).

### Summary of biopsy results

Biopsy results confirmed the absence of infection in the negative control group (Group 1).

In Group 8 the two sheep that progressed to clinical onset at 1093 and 1673 days were positive at biopsy at 558 and 1252 days respectively. The one animal in Group 9 that did not progress to clinical onset was negative at biopsy at 1484 days, 310 days before it was culled, and also at necropsy (Table 1).

## Discussion

The design of this study was originally intended to enable comparison between experimental exposure groups and the positive control Groups 5 to 7, which experienced exposure typical of that in VLA scrapie flock, using survival times and attack rates as indicators of the infectious dose received. We found that attack rates were not a reliable indicator of the infectious dose received since the majority of exposed animals became affected. In addition, survival times for VRQ/VRQ lambs born into the VLA scrapie flock in 2002 and 2003 were significantly different, probably due to the removal of a proportion of pre-clinically infected animals to service other projects, so it was not possible to directly compare survival times across all experimental groups. Therefore, survival analysis techniques were used to determine whether or not there were significant differences in risk between groups (Table 2, Figure 1).

The VRQ/VRQ model described in this study would appear to represent a worst case scenario, since the experimental conditions under which this study was conducted were extreme and involved an incidence of scrapie that could never be sustained in commercial flocks. The low incidence of scrapie in ARQ/ARQ sheep in the VLA scrapie flock, despite its establishment from flocks that included confirmed scrapie cases of that genotype, highlights the complexity of adopting a 'one size fits all' policy for the control of scrapie, and of the difficulties of accommodating variability arising as a result of genotype.

Despite the difficulties presented by the differences in risk of infection between 2002 and 2003 birth cohorts and the high attack rates in most exposure groups, this study has confirmed the fact that in this particular flock no part of the management cycle can be considered 'risk-free'. Our results support the conclusions of modelling of transmission in the INRA flock in France [23], where transmission appeared to occur seasonally in association with lambing, but the occurrence of horizontal transmission at other times could not be ruled out.

In line with historical evidence [7,16,23-25] it would appear that lambing and the lambing environment represent a substantial source of infectivity.

There was a trend for the positive control sheep (Groups 5-7) to survive longer than those in the 2002 VLA scrapie flock cohort, although this was only significant for Group 6. The survival times of Groups 5-7 were not significantly different which indicates that lambs in all three groups were exposed to sufficient infectivity in the first 6 weeks of life to induce clinical disease, without the need for long term exposure to the lambing pens or the flock as a whole.

It is important to recognise that lambs in Groups 5-7 and the VLA scrapie flock cohorts were potentially exposed to maternal as well as horizontal infection. Not all dams survived to the point where a positive post-mortem diagnosis of scrapie was possible but there was considerable variation in the survival times of Group 5-7 dams from lambing until death or culling (data not shown), suggesting that the dams may have been at different stages of incubation at the time that the groups were established.

Historical evidence for long term contamination of farms is derived primarily from Iceland, where restocking with sheep from scrapie-free areas frequently led to recrudescence many years later [26,27]. Farm buildings have been suggested as the most likely source of infectivity after restocking [26].

We found that exposure to a contaminated lambing environment provided sufficient infectivity to cause disease. Survival times for Group 4 and the 2003 VLA scrapie flock cohort were not significantly different, clearly demonstrating that neither contact with the affected flock nor exposure to contaminated pasture is necessary to induce clinical disease with similar survival times. Furthermore, subsequent direct contact with infected sheep and/or pasture does not significantly reduce the time taken to develop clinical disease. This is supported by the similar survival times observed in Groups 3 and 4 and the 2003 VLA scrapie flock cohort.

Increased risk of scrapie has previously been reported in flocks that always lamb at the same location compared to flocks that periodically vary the site of lambing, and in flocks that lamb in groups rather than in individual pens [16]. It has also been shown that failure to recover the placenta from the lambing pen is associated with increased odds of scrapie-positive status [25]. This has implications for scrapie-affected flocks where ewes are housed or confined in the same place for prolonged periods during lambing time, rather than the 'lamb, mother-up and out to pasture' management used in this flock.

We have shown that pasture remains infectious for at least 36 days after the removal of infected sheep and that exposure to contaminated pasture alone can also result in infection and clinical disease. Survival times of sheep in Group 10 and Groups 8 and 9 were significantly longer than the 2002 and 2003 VLA scrapie flock cohorts respectively. This suggests that although exposure to contaminated pasture is sufficient to cause disease, additional exposure to infectivity at lambing and via contact with infected sheep increases the risk of developing disease.

Due to the difference between the 2002 and 2003 VLA scrapie flock cohort survival times we were not able to investigate the possible dose response effect across Groups 8-10. However, the attack rate for Group 8 was lower than that for Group 9. This implies that when sheep are exposed to low levels of infectivity on pasture increasing the exposure period may increase the risk of developing disease.

It was not possible to demonstrate a statistically significant dose response relationship for Groups 11-13, which grazed with the VLA scrapie flock for varying lengths of time, which suggests that transmission occurs easily and early in the first six weeks of life, when the lambs are putatively most susceptible [28,29].

Healy and colleagues [25] found that spreading of sheep compost containing manure and/or placenta onto pasture was associated with increased odds of a flock being scrapie-positive. It remains to be determined whether pasture contamination is persistent and associated with grass or soil. The difference in behaviour of the 2002 and 2003 VLA scrapie flock cohorts may imply that environmental contamination is most effective in the presence of sheep that are at, or close to, clinical onset.

The effect of contact with infected sheep after the peri-natal period was investigated by pair-wise comparison of Group 8 and 11, and Group 9 and 12 (Table 3). In both cases the survival times were longer and the attack rates lower for Group 8 and 9, indicating that direct exposure to infected animals increases the risk of developing clinical disease. It was not possible to directly compare Group 10 and 13 since lambs were born in different years.

The longer survival times observed in Groups 5-7 compared to the 2002 VLA scrapie flock cohort are most probably due to additional factors that affect sheep after 12 months of age.

Sheep in Groups 5-7 were exposed both to the infected flock and contaminated pasture for 8-18 months less than those in the 2002 VLA scrapie flock cohort, which remained in the flock until clinical end-point. Therefore the lifetime exposure levels of the 2002 VLA scrapie flock cohort would have been higher than for Groups 5-7, although dose-related differences between the groups may be obscured by the small group size. The length of the incubation period may be due to cumulative exposure (total dose of infectivity model) or to an independent combination of individual challenges. Analysis of multiple low dose challenges in experiments with rodents [30] suggested that although repeated doses lead to an increased risk of infection, incubation periods are inconsistent and shorter than expected. Challenge using multiple sub-optimal aliquots of a fixed dose, rather than a single large dose, significantly reduces the probability of infection [30].

In addition, disease progression may be accelerated by stresses associated with husbandry environment and/or breeding. It could be argued that the sheep which spent most of their lives at the High Mowthorpe containment facility, housed indoors with familiar pen-mates and fed a pre-mixed feed of consistent quality, were subject to fewer environmental and nutritional stresses that the VLA scrapie flock sheep. Sheep in the VLA scrapie flock spend most of the year outdoors at pasture and sheep are routinely added (home-bred lambs and animals purchased to maintain genotype diversity) and removed from the flock to meet VLA and external project demands. With regards to breeding: none of the experimental groups withdrawn to clean premises were mated, while the majority of the 2002 VLA scrapie flock cohort would have lambed or been in lamb when they developed clinical disease.

The mechanisms of transmission from sheep to sheep, or of pasture contamination in the absence of lambing, remain to be determined. As demonstrated in laboratory models, faecal [31] or urinary contamination [32] are the most obvious candidates, and potentially supported by evidence of immunolabelling of disease-associated prion protein (PrP^d^) in the kidneys of scrapie affected sheep [33,34]. In a recent study involving the experimental infection of scrapie-free lambs by feeding with milk from scrapie-affected ewes [29] there was also evidence of horizontal transmission between lambs infected through consumption of milk and control lambs in the post-exposure period. This may imply that transmission between lambs is a component of natural epidemiology that has previously been unrecognised, and could have been responsible for smoothing out differences between groups in this study where exposure levels were otherwise low and presumed to have been from a single source, such as pasture.

This study made no attempt to directly quantify the amount of infectivity present in any part of the farm environment. PrP^d ^appears to bind closely to soil [35,36] and by doing so appears to become more infectious, at least to hamsters, than when fed un-bound. Invertebrates have also been postulated as potential reservoirs of infectivity on pasture, or in the farm environment [37,38], but their involvement in the epidemiology of scrapie has not yet been conclusively demonstrated. Whether or not they play a role, it seems clear that direct contact with scrapie-infected sheep increases the risk of transmission to other sheep.

## Conclusion

The confirmation that all parts of the farm management cycle represent a risk supports past epidemiological assumptions. Risks are not equal at all times in the management cycle: it is a multifactorial problem in which the contribution of different parts of the management cycle may vary. Contact with infected animals is important and should be minimised. Decontamination of entire farm premises as part of control measures is clearly impractical. Our results suggest that decontamination should focus on contaminated buildings or/and the avoidance of further use of fields that have been used repeatedly for lambing.

The low incidence of scrapie in ARQ/ARQ sheep in the resident flock confirms the importance of genotype selection for the control of scrapie, in the knowledge that in some flocks only ARQ/ARQ sheep are affected. Variability in susceptibility between genotypes may also correlate with the strain of scrapie circulating in the flock. Consequently, repopulation of depopulated farm premises must take into account such factors with a view to minimising the risk of recrudescence.

Investigation into the prevalence of sub-clinical and clinical infection in a flock may assist in determining the extent to which decontamination is required, and whether genetic selection of replacement stock may suffice in ensuring that they remain free of infection.

## Methods

The study design, described in detail below, was intended to enable comparison of attack rates and incubation period in groups of lambs born to naturally infected ewes with those in lambs born to TSE-free ewes, but exposed to parts of the flock cycle in the naturally infected flock, for different periods of time. Groups 5 to 7 represent the positive controls, born to infected ewes and removed after different periods of residence in the infected flock. While Group 2 experienced exposure to all parts of the management cycle for a full year, Group 3 was only exposed to infected ewes at lambing and remained in the lambing pens for a short period after the infected sheep were removed to pasture. Group 4 on the other hand had no contact with infected sheep, being exposed only to pens previously used for lambing. Groups 11-13 came into contact with the infected flock only at pasture, for varying lengths of time, while Groups 8-9 had no direct contact with sheep, but grazed pasture previously grazed by the infected flock, again for varying lengths of time. The expectation was that differences in attack rates and incubation would reflect exposure levels, determined in part by the location of exposure with presumed variability in levels of contamination, and partly by length of exposure.

### The Scrapie-affected flock

A naturally infected but experimental flock of sheep maintained by the VLA and its farm environment provided the source of scrapie infectivity to which the scrapie-free sheep were exposed. Lambs of the VRQ/VRQ genotype born into this flock always develop clinical disease, usually at around 24 months of age. Consequently, this study focused on lambs of the VRQ/VRQ genotype in order to maximise the likelihood of transmissibility and accumulation of data with the minimum of delay.

All procedures involving animals were approved by the Home Office under the Animals (Scientific Procedures) Act 1986, and the ethics committees of the VLA and Agricultural Development and Advisory Service (ADAS).

The VLA scrapie flock was established in 1998 through the purchase of siblings and offspring of various breeds and genotypes born to scrapie-infected dams from breeding and commercial flocks. The sheep were subsequently managed conventionally although natural mating, embryo transfer and artificial insemination are utilised for breeding. The breeding programme for this flock aims to ensure a continued population of fully susceptible genotypes in order to maintain a high infection pressure.

The sheep spend most of the year at pasture but ewes are brought indoors in late winter and housed in large groups on deep straw for one to two months prior to lambing. After lambing ewes are individually penned with their lambs for a few days to promote good mothering. Ewes and lambs are put out to pasture on a weekly basis when the lambs are approximately one week old, and follow a pasture rotation grazing regime dependent on the availability of grass. Lambs are naturally weaned at 12 weeks of age and then maintained as a group on pasture until they join the main flock in the autumn.

### The TSE-free sheep

The TSE-free ewes and lambs of the Cheviot breed used in this study originated in a VLA flock derived from sheep imported from New Zealand. This flock is located at a remote establishment where all aspects of its management are designed to ensure that its TSE-free status is maintained [39].

### Experimental Groups

This study was designed to coincide with the normal spring lambing of the VLA scrapie flock. TSE-free ewes carrying VRQ/VRQ genotype lambs were synchronised to lamb at the same time as the VLA scrapie flock. Lamb availability (5 or more per group) required the study be extended over two lambing seasons. The experimental groups are summarised in Table 1.

Lambs born to ewes from the VLA scrapie flock and carrying the VRQ/VRQ genotype were used to establish positive control Groups 5, 6 and 7. For these three groups only the breeds were not restricted to Cheviot (n = 7), but included Swaledale (n = 2) and Welsh Mountain (n = 5) breeds. All dams were VRQ heterozygotes since in this flock VRQ homozygotes succumb to disease before they are old enough to lamb. Their lambs were reared in the infected environment according to normal management conditions before removal to clean barrier accommodation at High Mowthorpe at six weeks, three months and twelve months of age respectively. To prevent cross contamination, once in the barrier accommodation all groups were kept separate from each other.

To establish Groups 2 and 3, TSE-free pregnant ewes were transported from their original farm to clean premises at the VLA, and introduced directly into the lambing sheds with the VLA scrapie flock two weeks prior to lambing. They were housed with the VLA scrapie flock in-lamb ewes until they lambed. Group 2 went to pasture with the VLA scrapie flock after lambing, while Group 3 remained in the lambing accommodation for six weeks and were moved directly to clean barrier accommodation without further exposure to pasture or infected sheep.

Group 4 was established by introducing TSE-free dams into the lambing environment after the removal of the VLA scrapie flock ewes. The lambing shed had previously held a group of ewes that lambed down over a period of twelve days. The TSE-free ewes lambed within forty-eight hours and remained in the lambing shed for six weeks before removal with their lambs to barrier accommodation.

The TSE-free dams that supplied Groups 1 and 8-13 lambed at the clean VLA premises prior to their introduction to the appropriate exposure environment. For Groups 8-13 this occurred when the lambs were two days of age.

Groups 8, 9 and 10 investigated the effect of exposure to pasture grazed by the VLA scrapie flock, following removal of the infected flock at least 36 days before the introduction of the TSE-free sheep. The experimental sheep followed the VLA scrapie flock sheep as they moved from paddock to paddock under the normal management regime. At no time was the gap between departure of the infected flock and introduction of the lambs less than 36 days. Lambs were removed at six weeks (Group 8), three months (Group 9) and twelve months (Group 10) post-introduction. Group 10 was at pasture in 2002 while Groups 8 and 9 were at pasture in 2003.

The effect of direct contact with the scrapie-infected ewes and their lambs at pasture (Groups 11, 12 and 13) was investigated by introducing the TSE-free dams and their lambs to the VLA scrapie flock at pasture. These groups of lambs were removed to the barrier accommodation at six weeks, three months and 12 months respectively.

The negative control ewes and lambs, Group 1, remained in the VLA clean lambing environment until the lambs were six weeks of age, before removal to the barrier accommodation. All lambs remained with their dams until weaning at 12 weeks of age, but after separation the study did not include investigation of the infection status of the dams.

All lambs were ear-tagged soon after birth and their tails docked using the rubber ring method. Male lambs were similarly castrated. Navels were treated with iodine solution. Subsequent routine husbandry treatments, for example foot trimming and drenching, were organised to ensure that groups not in direct contact with the scrapie-affected sheep were treated before any potentially infected sheep. Single-use needles were used when injectable substances were administered.

The lambs, segregated from other groups in their barrier accommodation, were monitored daily for signs of clinical disease and weighed monthly. Tonsil, palpebral and/or rectal tonsil biopsies [22,40] were collected periodically with single use disposable instruments from some, but not all, groups in order to assist in interpretation of infection status if the clinical end point was not reached. All biopsies were examined by immunohistochemistry as described previously [22,40].

Animals were euthanized using quinalbarbitone sodium (Somulose, Arnolds) once the standardised clinical end point was reached (exhibiting pruritus for 60% of a day or with lesions arising due to continuous rubbing). Although the brain and selected visceral tissues were collected at necropsy to facilitate further study (if required), only the medulla at the level of the obex was subjected to immunohistochemical examination as described previously [41].

Because of the need to establish the experimental groups over a two year period, lambs of the VRQ/VRQ genotype born into the VLA scrapie flock in 2002 and 2003 were included in the final analysis to compensate for variability in infection pressure from year to year. The VLA scrapie flock experienced clinical scrapie in sheep of other genotypes in both years, particularly in ARQ/VRQ and to a lesser extent in ARQ/ARQ sheep, and these will have contributed to the environmental contamination. Nevertheless, only VRQ/VRQ sheep were considered to be appropriate indicators of risk for direct comparison with the study groups.

### Statistical analysis

Data was recorded and stored in a Microsoft Access database. The statistical software programme STATA 8 (StataCorp 2003. Stata Statistical Software Release 8.0 College Station, TX: Stata Corporation) was used for the statistical analysis.

Initial descriptive analysis was followed by exploration of the data using Kaplan-Meier survival plots. Cox proportional hazards models were then fitted to the data to compare the survival of the experimental groups. The outcome (failure) event was death due to clinical scrapie and the baseline for comparison was the relevant natural birth cohort of the VLA scrapie flock. Intercurrent deaths before the clinical end point were included in the analyses as censored observations. The likelihood ratio test was used for testing hypotheses concerning more than two experimental groups and the Wald test for comparing pairs of groups (Table 3), both with *P *= 0.05 as the threshold level for statistical significance.

## Authors' contributions

GD led the project through most of its time-course and managed live animal monitoring and post-mortems. ST advised on appropriate analyses and interpretation arising from prior epidemiological characterisation of the resident flock. LH was project manager for barrier accommodation facility. AD and SJM conducted all examinations of biopsy and post-mortem samples, supervised by MMS. RS conducted the statistical analyses. HS co-ordinated the breeding and transfer of the experimental sheep to barrier accomodation. DM had overall responsibility for delivery of the project, and led in the drafting of the manuscript. All co-authors participated in the drafting of and have read and approved the final manuscript.
